# Can We Reliably Calibrate Deep Nodes in the Tetrapod Tree? Case Studies in Deep Tetrapod Divergences

**DOI:** 10.3389/fgene.2020.506749

**Published:** 2020-10-16

**Authors:** Jason D. Pardo, Kendra Lennie, Jason S. Anderson

**Affiliations:** ^1^Department of Comparative and Experimental Biology, Faculty of Veterinary Medicine, University of Calgary, Calgary, AB, Canada; ^2^McCaig Institute for Bone and Joint Health, University of Calgary, Calgary, AB, Canada; ^3^Department of Biological Sciences, University of Calgary, Calgary, AB, Canada

**Keywords:** tetrapod, prior constraint, node age prior, fossil record bias, phylogeny

## Abstract

Recent efforts have led to the development of extremely sophisticated methods for incorporating tree-wide data and accommodating uncertainty when estimating the temporal patterns of phylogenetic trees, but assignment of prior constraints on node age remains the most important factor. This depends largely on understanding substantive disagreements between specialists (paleontologists, geologists, and comparative anatomists), which are often opaque to phylogeneticists and molecular biologists who rely on these data as downstream users. This often leads to misunderstandings of how the uncertainty associated with node age minima arises, leading to inappropriate treatments of that uncertainty by phylogeneticists. In order to promote dialogue on this subject, we here review factors (phylogeny, preservational megabiases, spatial and temporal patterns in the tetrapod fossil record) that complicate assignment of prior node age constraints for deep divergences in the tetrapod tree, focusing on the origin of crown-group Amniota, crown-group Amphibia, and crown-group Tetrapoda. We find that node priors for amphibians and tetrapods show high phylogenetic lability and different phylogenetic treatments identifying disparate taxa as the earliest representatives of these crown groups. This corresponds partially to the well-known problem of lissamphibian origins but increasingly reflects deeper instabilities in early tetrapod phylogeny. Conversely, differences in phylogenetic treatment do not affect our ability to recognize the earliest crown-group amniotes but do affect how diverse we understand the earliest amniote faunas to be. Preservational megabiases and spatiotemporal heterogeneity of the early tetrapod fossil record present unrecognized challenges in reliably estimating the ages of tetrapod nodes; the tetrapod record throughout the relevant interval is spatially restricted and disrupted by several major intervals of minimal sampling coincident with the emergence of all three crown groups. Going forward, researchers attempting to calibrate the ages for these nodes, and other similar deep nodes in the metazoan fossil record, should consciously consider major phylogenetic uncertainty, preservational megabias, and spatiotemporal heterogeneity, preferably examining the impact of working hypotheses from multiple research groups. We emphasize a need for major tetrapod collection effort outside of classic European and North American sections, particularly from the southern hemisphere, and suggest that such sampling may dramatically change our timelines of tetrapod evolution.

## Introduction

Modern biodiversity is generally organized into large, relatively ancient, clades (i.e., Amniota, Mammalia, and Reptilia) with characteristic body plans and broad ecomorphological similarity. Building a comprehensive understanding of the origin and diversification of these major taxa is a uniquely challenging research program. Often, we are studying groups that originated long ago, defined by long branches to living representatives of the clade and at its base. For example, crown Tetrapoda (the most recent ancestor of living reptiles, mammals, and amphibians) diverged from its most recent living clade, the lungfish (Takezaki and Nishihara, 2017) over 400 million years ago (e.g., Zhu and Yu, 2002), leaving a long stem occupied by a diversity of fossil species that document important evolutionary events such as the acquisition of limbs and digits and emergence on land. As a result, these major taxa are often quite distinct from their closest living relatives, making it difficult to isolate specific intrinsic and extrinsic drivers that may explain their success. Intrinsic factors typically refer to heritable factors that govern a population’s ability to generate new forms through evolutionary novelties, changes in evolvability, and developmental canalization (Hendrikse et al., 2007) or ability of established forms to maximize fitness in a range of possible environments through ecology, physiology, plasticity, and functional morphology (Schluter, 2000). Extrinsic factors, on the other hand, typically refer to large-scale changes in the overall state of the Earth’s biosphere, including changes in biogeographic connectivity due to plate tectonics (San Mauro et al., 2005; Pyron, 2014), shifts in nutrient or oxygen availability (Ward et al., 2006), shifts in global climate (Chaboureau et al., 2014), and global mass extinction events that either serve as discrete events, which culled global diversity (Raup and Sepkoski, 1982; Sallan and Coates, 2010) or vacated niches to permit subsequent diversification of survivors (e.g., Field et al., 2018). Given that these hypothesized extrinsic factors explicitly invoke geological or macroecological conditions that existed at a specific time in Earth’s history, testing a relationship between these factors and the evolution of major taxa requires precise, accurate constraints on the timing of the origin and diversification of those taxa.

As the fossil record is incomplete, it is often impossible to directly use fossils to establish tight constraints on the origin of major taxa. To address this problem, a series of methods have been devised to use relative difference in molecular sequence between two taxa to estimate the age of the divergence between those taxa. These methods, collectively termed “the molecular clock,” integrate paleontological data (as node calibration dates) and molecular data (as sequence divergence or estimated branch length) to produce estimates of the ages of all nodes on a phylogenetic tree. Although early implementation of these methods was highly procedural and prone to multiplication of error (Graur and Martin, 2004), newer approaches have re-envisioned node calibration dates as a range of prior probabilities for the age of a node (“node priors”), allowing coestimation of tree topology and age (Stadler and Yang, 2013), potentially improving precision of node estimates. Furthermore, a series of *a posteriori* methods have been created to assess quality of individual node calibrations within a set of calibrations. In these approaches, the quality of individual calibrations is tested by comparing how well each calibration can predict the ages of all other calibrations (Near and Sanderson, 2004; Stadler and Yang, 2013; Heath et al., 2014). Integration of these methods into phylogenetic analysis has even been used as a means of discerning between phylogenetic trees (Lee and Yates, 2018; King and Beck, 2019) and for dating the age of specific fossils for critical evaluation (King and Beck, 2019). These methods are appealing because practitioners are free to engage with mathematically tractable patterns in the data rather than engage in taxonomic arguments of otherwise narrow interest, based on broadly inaccessible and subjective debates on the importance of specific anatomical features for inference of phylogeny. Conversely, these analytical approaches effectively give hypotheses of rate of change veto power over the estimated fossil age and taxonomic ID that serve as the primary data used to test those hypotheses. This has led to an emerging analytical pipeline that selects trees or calibration ages *a posteriori*, and in doing so excludes or reinterprets primary data that inconveniently conflicts with the overall pattern of results (King and Beck, 2019). Although the long-term utility of these methods remains to be seen, *a priori* assessment of the quality of *a priori* node calibrations must retain logical primacy in assessing the quality of a molecular clock (Hedges et al., 2018; Morris et al., 2018).

Node-age calibrations themselves require a detailed assessment of the fossil record to identify the earliest member of a given clade. Identifying the earliest members of a clade requires substantial specialist knowledge of the anatomy of the group, how variation in that anatomy corresponds with the crown group, and the temporal distribution of fossils that exhibit that anatomy. This specialist knowledge from paleontology is often far outside the expertise of molecular phylogeneticists. To facilitate easy access to this knowledge, compendia of node calibration dates have been assembled first by Benton and Donoghue (2006) and more recently by Benton et al. (2015). These compendia present a list of node minima and maxima for many clades in the tree of life and typically claim a lack of ambiguity over these proposed node calibration ages. These compendia are widely treated as expert-vetted calibration points in molecular clock studies (Feng et al., 2017; Hime et al., 2020), with little to no direct consultation with experts. This assumes several things: that paleontological experts address phylogeny in a manner consistent with usage by molecular clock approaches, that compendia such as Benton et al. (2015) accurately report consensus between paleontological workers and stability of the underlying tree, and that stability of age estimates reflects biological processes recorded in molecular data.

To date, discussions refining best practices in node calibration have focused on ensuring that fossils chosen as node-age calibrations fall with certainty within the crown group, that their precise stratigraphic resolution is provided, and that this precise stratigraphic resolution is placed into an explicit numerical framework (Parham and Irmis, 2008; Parham et al., 2012). However, considerably less attention has been given to factors influencing calibrations of deeper nodes indicating the divergence of major clades. These nodes are important because they often serve as external bounds on node age interpolation (Duchêne et al., 2014) and because their position deep within the tree of life means they are likely to appear frequently in studies using a molecular clock (Müller and Reisz, 2005; Chen et al., 2015; Feng et al., 2017; Hime et al., 2020). Given the importance of reliable node age calibrations in these deeper nodes, it is critical to ask whether current recommendations of best practices, and the calibrations outlined in compendia, are sufficient.

Three such nodes of interest are the deep divergences within the Tetrapoda. Tetrapoda is a monophyletic grouping that includes all descendants of the common ancestor of modern amphibians, reptiles, birds, and mammals. These make up the entirety of extant vertebrates with digited limbs. The term “Tetrapoda” is generally applied to digited members of the total group, a usage that is equivalent to Stegocephalia (Laurin et al., 2000), whereas members of the crown-group are sometimes referred to Neotetrapoda (Sues, 2019). Tetrapoda consists of two clades: the Lissamphibia and Amniota (Chen et al., 2015; Irisarri et al., 2017; Hime et al., 2020). Lissamphibians include the caecilians (Gymnophiona), frogs (Anura), and salamanders (Caudata) and are characterized by thin, permeable mucous skin. Amniota includes mammals (Mammalia), birds (Aves), and ‘reptiles’, and is characterized by keratinized skin and a unique extraembryonic membrane, the amnion (Reisz, 1997). Each of these clades is notable in that they are all very old (>265 Ma) and that the monophyly of each clade is not in serious contention (Chen et al., 2015; Irisarri et al., 2017; Hime et al., 2020). Additionally, in each case modern body plans are extremely different from fossil forms, to the extent that it is difficult if not impossible to identify diagnostic characters of the crown group without reference to fossil diversity.

We here review these three calibration points to understand how current best practices for node calibration may fail to guide calibration of Palaeozoic nodes. We discuss how phylogenetic problems in the Palaeozoic, including node calibration, are almost entirely dependent on interpretation of morphology among fossil groups rather than reference to an independently inferred molecular phylogeny. We then explore specific features of phylogenetic uncertainty among Palaeozoic tetrapods, and how subtly different interpretations of Palaeozoic tetrapod interrelationships suggest very different timelines for the origin of these three tetrapod clades. We finally discuss general spatial and temporal patterns in the early tetrapod fossil record, and how these may bias against discovery of early members of each clade. Finally, we provide recommendations that we believe will mitigate some of the problems currently affecting these node calibrations and that may provide a framework for efforts to calibrate similar nodes in other taxa.

## Origins Versus Affinities

Assigning a node age calibration requires identification of the oldest known fossil that can be assigned to an extant clade, but there is some variation in how this is done. To establish universal standards, Parham et al. (2012) outlined a set of best practices. This set of best practices focuses largely on connecting an occurrence with stratigraphic information. Less attention has been given to outlining standards for ensuring that the fossil used in a calibration in fact belongs to the clade in question; Parham et al. (2012) suggest that apomorphies be identified in the specimen used to date the clade, but do not suggest universal standards for how these apomorphies are to be chosen in the first place.

How are these apomorphies chosen in practice? To examine this, we use as an example only the calibration list of Hime et al. (2020), a recent phylogenomic analysis of lissamphibian diversification employing a molecular clock. This calibration list is chosen here as it represents one of the larger and more comprehensive calibration sets employed across early vertebrate diversity, and is largely consistent with other recent calibration sets, such as Cannatella (2015) and Feng et al. (2017). Hime et al. (2020) employ a total of nineteen calibration points. Of these, the oldest calibration point (Tetrapoda) has a minimum age of 337 Ma, whereas the youngest minimum calibration point (*Ptychadena* + *Phrynobatrachus*) is set at 25 Ma. The methodologies for choosing these calibration points are varied; several taxa (*Chunerpeton tianyiensis* and *Iridotriton hechti*) were initially assigned to nodes without an explicit phylogenetic analysis (Gao and Shubin, 2003; Evans et al., 2005), and two taxa (*Calyptocephalella pichileufensis* and an unnamed fossil ptychadenid) have never been assessed within a phylogenetic framework (Gómez et al., 2011; Blackburn et al., 2015). In these cases, assignment to a given clade is accomplished solely through comparative anatomy and reference to differential diagnoses. However, most nodes have been assessed through some manner of phylogenetic analysis. In the case of all node calibrations in the Mesozoic and Cenozoic, these phylogenetic analyses invariably include at least a subset of extant taxa. In fact, these analyses typically include a large majority of extant taxa, but nodes representing divergences in the Paleozoic differ in the constitution of the overall phylogenetic sampling. Phylogenetic analyses cited for node calibrations of the divergence of Amniota, Batrachia, Lissamphibia, and Tetrapoda do not sample a single extant taxon in any cited case (see Anderson, 2008 for further discussion).

This distinction between Paleozoic and post-Paleozoic divergences is noteworthy. Relationships of fossils used as node calibrations in the Mesozoic and Cenozoic are investigated via comparison with the specific taxa sampled for molecular sequence data, and interrelationships between fossil taxa are generally not important for resolving phylogenetic disputes. In direct contrast, node calibrations in the Palaeozoic depend on fine interrelationships between sometimes-obscure fossil taxa with little to no direct comparison with extant organisms. This places molecular phylogeneticists in a predicament: calibration of Paleozoic nodes may require engagement with paleontological literature and contending with disputes among those workers.

## Node Minima: What Are the Earliest Representatives of the Major Tetrapod Crown Groups?

When we talk about phylogenetic uncertainty of fossils involved in node calibration, we typically have in mind a situation where there is a relatively dense phylogeny of modern taxa and the difficulty is in finding fossils that preserve sufficient diagnostic anatomy to be placed confidently into this phylogenetic framework (Patterson, 1981; Parham et al., 2012). In a situation such as this, diagnostic characteristics can be determined *a priori* through comparative anatomy of extant organisms with known phylogenetic relationships. In such an ideal case, the primary challenges are local uncertainty in phylogeny and in specific node age calibrations, and many tools, such as Bayesian tip-dating (Stadler and Yang, 2013) are designed to handle these problems by assessing these sorts of local patterns of uncertainty (uncertainty of local tree resolution, uncertainty of specific fossil age interpretations) as a range of posterior probabilities. Under these circumstances, molecular phylogeneticists do not need to engage with the paleontological record beyond identifying the taxa that need to be incorporated into an analysis.

**FIGURE 1:**
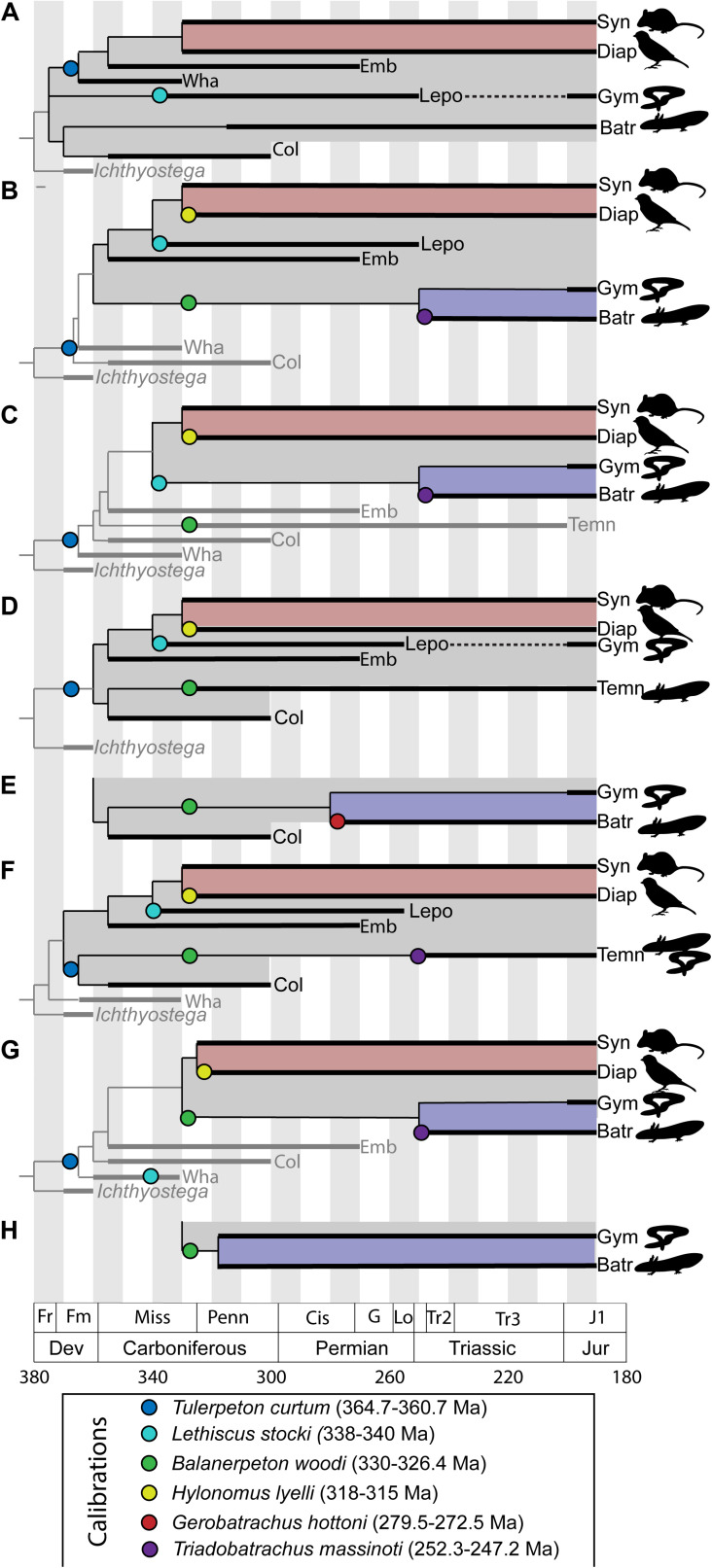
Comparative phylogenetic hypotheses of early tetrapod interrelationships and lissamphibian affinities, showing effects on node calibrations for the age of tetrapod (gray) and lissamphibian crown (blue). **(A)** Modified from Carroll (2001); **(B)** modified from Ruta and Coates (2007); **(C)** modified from Vallin and Laurin (2004); **(D)** modified from Anderson et al. (2008); **(E)** variant of **(D)** modified from Maddin et al. (2012); **(F)** modified from Clack et al. (2017); **(G)** modified from Pardo et al. (2017a); **(H)** variant of **(G)** modified from Pardo et al. (2017b). Taxonomic abbreviations: Batr, Batrachia; Col, Colosteida; Diap, Diapsida; Emb, Embolomeri; Gym, Gymnophiona; Lepo, Lepospondyli; Syn, Synapsida; Temn, Temnospondyli; Wha, Whatcheeriida. Gray boxes delineate the tetrapod crown, red boxes delineate the amniote crown, and blue boxes delineate the lissamphibian crown (where present). Phylogenetic position of taxa often used to calibrate node ages denoted with colored circles.

**FIGURE 2:**
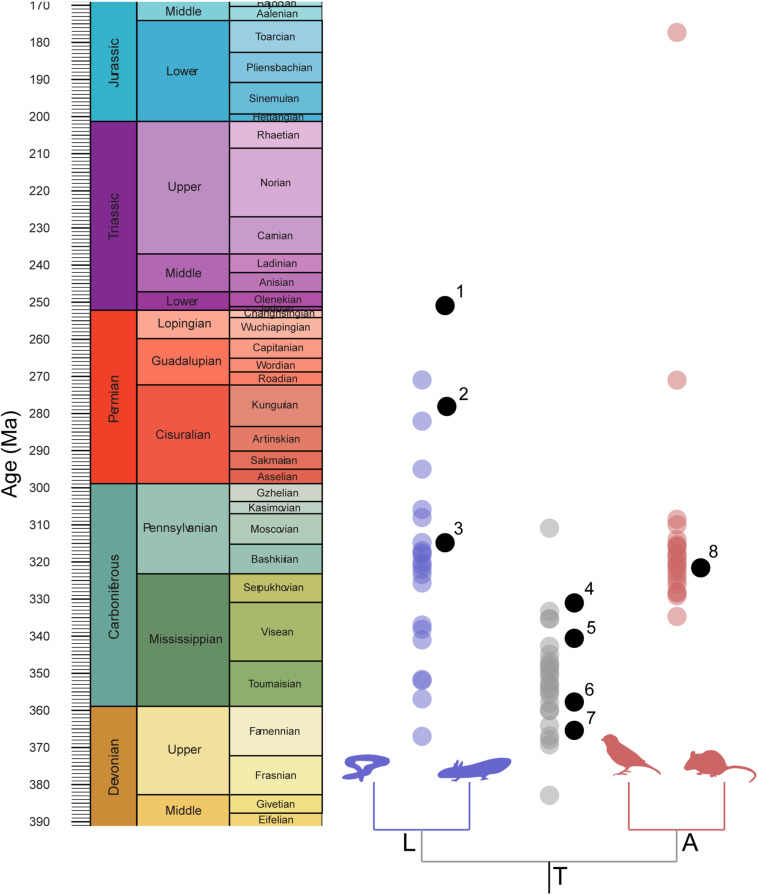
Distribution of implied node calibration ages compared with distribution of mean node-age estimates. Node estimates drawn from timetree.org. 1, *Triadobatrachus massinoti*; 2, *Gerobatrachus hottoni*; 3, *Amphibamus grandiceps*; 4, *Balanerpeton woodi*; 5, *Lethiscus stocki*; 6, Horton Bluff tetrapod fauna; 7, *Tulerpeton curtum*; 8, *Hylonomus lyelli*.

One way out of this problem has been for paleontologists to assemble compendia of recommended nodes for use in molecular clock calibrations and fossils to use for calibration of these nodes (Benton and Donoghue, 2006; Benton et al., 2015). These compendia provide lists of nodes and fossil taxa with reference to the paleontological literature, but generally do not provide substantial discussion of the specific bases for these attributions or differences in expert opinion. This approach is generally acceptable for Mesozoic and Cenozoic divergences, where the anatomical basis for relationships between modern groups is well-understood. However, this is not the case for deep divergences in the tetrapod tree. Early tetrapod phylogeny is highly unstable and lacking in consensus. Calibration of these nodes depends on broad anatomical comparisons across the entire early tetrapod diversification, beginning in the late Devonian and extending through the early Permian. These anatomical comparisons also extend to the earliest representatives of modern amphibian lineages in the Mesozoic (Maddin et al., 2012; Ascarrunz et al., 2016; Schoch et al., 2020), as these fossils preserve generalized tetrapod anatomy not seen in modern representatives of these groups and therefore provide insight into the relationships between amphibians, amniotes, and extinct tetrapod groups. This instability manifests as two major points of controversy: (1) what are the general interrelationships of major archaic early tetrapod taxa and (2) what is the relationship between major archaic early tetrapod taxa and lissamphibians? An addendum to the second point is that some workers have questioned the inclusiveness of the lissamphibian crown group itself, depending on how convergences between modern lissamphibian orders are interpreted (Anderson et al., 2008; Pardo et al., 2017b). Furthermore, the differences between these phylogenetic hypotheses are not trivial. Different phylogenetic hypotheses of early tetrapod relationships and of lissamphibian origins represent substantially different interpretations of the nature of crown tetrapod and crown lissamphibian characters, and a resulting different timeline of tetrapod origins (Figure 1). Given that phylogenetic analyses treating this problem must consider the anatomy of fossils spanning approximately the first 170 Ma of tetrapod evolution (Figure 2) and compare hypotheses suggesting very different patterns of body plan evolution, this is not a simple problem.

### Phylogenetic Context of the Amniote Crown

The phylogenetic relationships of vertebrate taxa associated with the origins of the amniote crown (i.e., the mammal-reptile split) are relatively stable (Laurin and Reisz, 1995). Amniotes are recognized as comprising two clades, the Reptilia and the Mammalia. Although some disagreement remains concerning the relationship of turtles among other reptiles (Chiari et al., 2012; Field et al., 2014; Bever et al., 2015; Schoch and Sues, 2015), there is essentially no disagreement concerning the monophyly of these two amniote clades. The phylogenetic relationships of modern amniote clades to Palaeozoic relatives are relatively stable, although some disagreements do exist.

The fossil record of total-group mammals (Synapsida) provides an exceptional record of the origin of the crown group from Palaeozoic ancestors. Broad trends in the assembly of the mammalian body plan has been reconstructed with wide consensus based on the dense record of total-group mammals (therapsids) from the late Permian and early Triassic, and confidently extending back through the Late Carboniferous “pelycosaurs” (Sidor and Hopson, 1998). These “pelycosaurs” can be assigned to several major clades, the Eupelycosauria, the Varanopidae, and the Caseasauria (Laurin and Reisz, 1995; Sidor and Hopson, 1998; Benson, 2012). Therapsids are thought to fall within the Eupelycosauria, whereas varanopids and caseasaurs are thought to represent successive outgroups to this clade (Sidor and Hopson, 1998; Benson, 2012).

The early record of synapsids has historically been relatively depauperate. The earliest definitive synapsid fossils are known from the Moscovian stage of the Carboniferous (315.2–307 Ma) of North America and the Czechia. These fossils are primarily attributable to eupelycosaurs, including *Archaeothyris florensis* (Reisz, 1972) and *Echinerpeton intermedium* (Reisz, 1972; Mann et al., 2019), although newly described fossils demonstrate the presence of a varanopid, *Dendromaia unamakiensis* from the same age (Maddin et al., 2020). Fragmentary fossils ambiguously attributable to synapids are known from the Bashkirian stage of the Carboniferous (320–315.2 Ma) of Joggins, Nova Scotia. Bashkirian records of synapsids were previously limited to the partial skeleton *Protoclepsydrops haplous* (Carroll, 1964, although disputed by Reisz, 1972), but it has recently been proposed that *Asaphestera platyrhis*, previously considered a tuditanomorph “microsaur,” might in fact be a caseasaur synapsid from the same set of localities (Mann et al., 2020).

**FIGURE 3:**
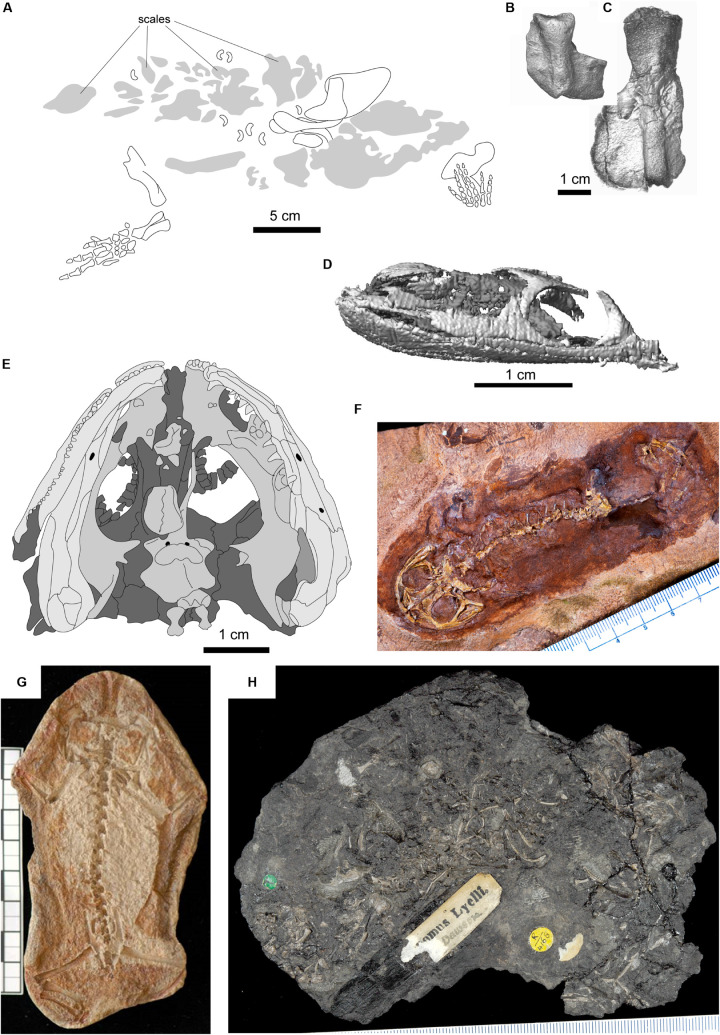
Selected fossils representing node-age calibrations. **(A)** *Tulerpeton curtum*, after Lebedev and Coates (1995); **(B)** Horton Bluff colosteid-like taxon; **(C)** Horton Bluff embolomere-like taxon; **(D)** *Lethiscus stocki*, segmented skull based on micro-CT; **(E)** type specimen of *Balanerpeton woodi*, after Milner and Sequeira (1993); **(F)** *Gerobatrachus hottoni*; **(G)** *Triadobatrachus massinoti*; **(H)** type specimen of *Hylonomus lyelli*.

The precise composition of the reptile stem group is somewhat more contentious. Earliest definitive members of the reptile crown group are relatively derived stem-archosaurs, such as the proterosuchids and prolacertids of the Permo-Triassic Boundary (e.g., Benton, 1984; Evans, 1984; Dilkes, 1998; Nasbitt, 2011; Ezcurra, 2016; Simões et al., 2018). A diverse assemblage of possible stem-reptiles (claudiosaurids, weigeltosaurids, and younginids) are known from the Upper Permian, but how these relate to Carboniferous and Early Permian taxa is uncertain. Traditional Carboniferous-Permian eureptiles have been assigned to four major groups: the Araeoscelidae, Protorothyrididae, Captorhinidae, and Parareptilia (Laurin and Reisz, 1995). Of these, the Araeoscelidae is thought to be most closely related to the stem-reptiles of the Upper Permian (Laurin and Reisz, 1995; Reisz et al., 2011; Ford and Benson, 2018) and protorothyridids are thought to represent a paraphyletic assemblage that includes both derived diapsid relatives, as well as early diverging captorhinids (Müller and Reisz, 2006). Phylogenetic treatments have variously found the parareptiles to be the sister clade of all other reptiles (Gauthier et al., 1988; Modesto et al., 2015), or slightly closer to the crown (less mesosaurs, Laurin and Reisz, 1995), or a polyphyletic assemblage of stem-reptiles, stem-turtles, or both (Bever et al., 2015; Laurin and Reisz, 1995; Modesto et al., 2015; Ford and Benson, 2020). Within this framework, the earliest hypothesized member of the reptile stem group is *Hylonomus lyelli* (Figure 2H) from the Bashkirian Joggins Formation of Nova Scotia (Carroll, 1964), with additional abundant reptile material preserved throughout the early Pennsylvanian (Figures 1, 3).

However, despite the appearance of phylogenetic consensus, there is in fact a large amount of uncertainty and disagreement on overall phylogenetic relationships of many of these archaic taxa with respect to the two major amniote clades. The overall framework of early amniote phylogeny, and therefore the actual phylogenetic affinities of fossils typically identified as earliest crown amniotes, depends largely on distinctions made by Carroll (1964) prior to systematic phylogenetic analytical techniques, validated in part by early phylogenetic analyses (e.g., Laurin and Reisz, 1995). Internal organization of early divergences within Amniota has varied across a number of analyses, including substantial reorganization of early synapsid relationships (Benson, 2012), a possible position of protorothyridids at the base of the mammal stem (Brocklehurst et al., 2016; Matzke and Irmis, 2018), massive reorganization of early stem-reptiles (Laurin and Piñeiro, 2017; Ford and Benson, 2020), a paraphyletic Synapsida (MacDougall et al., 2018), and a possible displacement of a major clade of synapsids onto the reptile stem (Ford and Benson, 2018, 2020). This uncertainty suggests that attribution of fragmentary Bashkirian and Moscovian taxa, important for node date estimation, to either the reptile or mammal total groups may be volatile.

Furthermore, some taxa not traditionally considered amniotes have appeared within the Amniota in some recent analyses. Most notably, the Recumbirostra, a group of small fossorial tetrapods traditionally classified within a larger order Microsauria and sometimes considered to be related to extant amphibians (Vallin and Laurin, 2004; Marjanović and Laurin, 2013, 2019), has recently been placed on the reptile stem based on neurocranial similarities (Pardo et al., 2015, 2017b; Szostakiwskyj et al., 2015). This clade is relatively diverse in the Joggins Formation (Carroll, 1966; Mann et al., 2020), including forms such as *Steenerpeton sylvae*, *Trachystegos megalodon*, and *Leiocephalikon problematicum*, and *Hylerpeton dawsoni*, all of which can be confidently assigned to recumbirostran subclades for which complete articulated fossils exist. This contrasts with the relatively fragmentary fossils attributed to *Hylonomus lyelli* (Figure 3H; Carroll, 1964), as well as the fragmentary and ambiguous fossils attributed to synapsids from the same locality (Carroll, 1964; Mann et al., 2020), allowing phylogenetic relationships to be assessed with greater confidence. Although recent work finding the recumbirostran diversification within the Amniota remains controversial (compare Pardo et al., 2017b with Marjanović and Laurin, 2019), recent studies continue to identify reptile-like anatomy of the recumbirostran temporal fenestra (Gee et al., 2019) and dentition (Gee et al., 2020). Additionally, the Diadectamorpha, traditionally conceptualized as the sister clade of amniotes, has been found within the mammalian total group on the basis of occipital morphology (Berman, 2000), a result that has recently received new support based on anatomy of the inner ear (Klembara et al., 2020). This latter result currently has no major implications for node calibrations, however, as the oldest diadectamorph fossils are substantially younger than most putative early crown amniotes.

### Phylogenetic Context of the Amphibian Crown

Extant amphibians can be assigned to three monophyletic orders, the frogs (Anura), salamanders (Caudata), and caecilians (Gymnophiona). Although there is some uncertainty about the interrelationships of these three groups (Marjanović and Laurin, 2013), most analyses support the existence of a Batrachian clade that comprised Anura and Caudata to the exclusion of Gymnophiona (Hime et al., 2020). Molecular analyses consistently recover an amphibian clade (Lissamphibia) to the exclusion of Amniota, but a minority of phylogenetic analyses of amphibian morphology have found Gymnophiona as the sister clade of Amniota (Figure 1D), thus rendering Lissamphibia polyphyletic (Carroll, 2007; Anderson et al., 2008), although this has been rejected in more recent iterations of those analyses (Maddin et al., 2012; Pardo et al., 2017a). The timing of the origin of the amphibian crown group is difficult to determine, in large part because the phylogenetic context of amphibian origins remains hotly debated. Earliest representatives of all three modern lissamphibian orders are already highly derived, making it difficult to define a lissamphibian bauplan, and this lack of a clear lissamphibian bauplan has subsequently led to difficulties in placing lissamphibians into Palaeozoic diversity more generally. Both classic comparative and modern analytical approaches to the phylogenetic relationships of lissamphibian origins have found relationships between modern lissamphibians and two groups of early tetrapods, the Temnospondyli and Lepospondyli. The former comprised mostly medium to large-bodied tetrapods with a few small-bodied lineages, but share features of the braincase, palate, and limbs with modern amphibians (Anderson et al., 2008; Sigurdsen and Bolt, 2009; Fröbisch and Shubin, 2011; Maddin and Anderson, 2012; Maddin et al., 2012; Witzmann and Werneburg, 2017), whereas the latter is mostly small-bodied and generally shares patterns of cranial bone reduction and vertebral consolidation with modern amphibians (Marjanović and Laurin, 2013, 2019). General trends in sequence heterochrony have been invoked in support of both phylogenetic hypotheses (Fröbisch et al., 2007; Olori, 2013; Laurin, 2019), but the implications of these data remain unclear. Among temnospondyls, most phylogenetic analyses place lissamphibians within amphibamid dissorophoids (Figures 1B,E). Phylogenetic analyses finding a lepospondyl origin of lissamphibians have typically placed lissamphibians within a clade that comprised “lysorophians” and brachystelechid “microsaurs” (Figure 1C), which are currently recognized by most workers as recumbirostrans as discussed above (Anderson et al., 2008; Maddin et al., 2012; Pardo et al., 2017b).

These alternative hypotheses have different implications for the age of the lissamphibian crown group, even though they primarily concern the nature of the lissamphibian stem group. The earliest unambiguous lissamphibian fossil is the stem-anuran *Triadobatrachus massinoti* (Figure 3H) from the earliest Triassic Sakamena Formation of Madagascar (Rage and Roček, 1989; Ascarrunz et al., 2016). Early caudates appear by the Middle Triassic of Kyrgyzstan (Schoch et al., 2020), whereas the earliest unambiguous stem-gymnophionans are Jurassic in age (Jenkins et al., 2007). In phylogenetic analyses that place lissamphibians within lepospondyls, no Palaeozoic tetrapods are found within the lissamphibian crown group (Marjanović and Laurin, 2013, 2019). Phylogenetic analyses that find lissamphibians within temnospondyls intermittently do find evidence of Palaeozoic representatives of the lissamphibian crown group, however. One possible Palaeozoic crown-group amphibian is the early Permian amphibamid *Gerobatrachus hottoni* (Figure 3F) from the Clear Fork Group (Kungurian) of Texas, which preserves a mosaic of anatomical features typical of anurans, caudates, and more generalized temnospondyls (Anderson et al., 2008). Different phylogenetic treatments have disagreed on the placement of *Gerobatrachus*, either placing it as the sister taxon to batrachians (Anderson et al., 2008; Maddin et al., 2012) or just outside the crown group (Sigurdsen and Green, 2011) in trees that align with the Temnospondyl hypothesis. However, the inclusiveness of the lissamphibian crown group depends more generally on the position of caecilians (Anderson, 2008). Most workers have not found evidence of Palaeozoic stem-group representation of gymnophionans. Pardo et al. (2017b) identified large-scale similarities between the caecilian skull and the skulls of a different temnospondyl group, the mostly Triassic-aged rhytidostean stereospondyls. Although similar levels of cranial consolidation between gymnophionans and specialized rhytidosteans may reflect convergence in headfirst burrowers, Pardo et al. (2017b) also identified a number of major anatomical similarities that cannot be so easily dismissed, suggesting that more inclusive phylogenetic analyses are necessary to properly test hypotheses of gymnophionan origins. If this phylogenetic hypothesis is correct, it would suggest a much more inclusive lissamphibian crown group and earlier origin of the amphibian crown group (Figure 1G). The earliest definitive crown lissamphibians in this phylogeny would be the dissorophoids *Amphibamus grandiceps* and an unnamed branchiosaurid from the early Moscovian Francis Creek Shale of Illinois, United States (∼315 Ma, Milner, 1982). The early temnospondyl *Eugyrinus wildi* from the Bashkirian (∼318–315 Ma) of the Lower Coal Measures Formation of Lancashire, United Kingdom, would be ambiguously assignable to the lissamphibian crown group as well (Milner, 1980).

### Phylogenetic Context of the Tetrapod Crown

Whereas the composition of the amniote crown group is relatively stable, and the composition of the lissamphibian crown group is only questioned in a minority of analyses, the composition of the tetrapod crown group among early tetrapods is hugely controversial with very little consensus (Ruta et al., 2003; Ruta and Coates, 2007; Anderson et al., 2008; Marjanović and Laurin, 2013, 2019; Clack et al., 2017; Pardo et al., 2017a, b). Because of substantial changes in understanding of early tetrapod phylogeny over the past 40 years, essentially every major group of Carboniferous tetrapods has been alternately placed both within the tetrapod crown group and outside of it in different phylogenetic hypotheses (Figure 1). Importantly, the temporal range of some of these groups appears to extend back in time to the latest Devonian, so these differences in phylogenetic hypotheses can have major implications on the minimum node calibration age for the tetrapod crown group. Discussions of phylogenetic uncertainty in the origin of the tetrapod crown group have attributed this uncertainty to one of two major problems: (1) that different hypotheses of lissamphibian origins imply a less inclusive (Lepospondyl hypothesis) or more inclusive (Temnospondyl hypothesis) tetrapod crown group within a more stable tetrapod phylogeny (Anderson, 2008; Marjanović and Laurin, 2013), or (2) that uncertainty of deep interrelationships between major Carboniferous tetrapod lineages stems from an explosive radiation dating back to the End Devonian mass extinction (Coates et al., 2008). Both factors contribute to overall uncertainty concerning the composition of the tetrapod crown group, although this appears to be a much broader problem.

As we noted above, paleontologists have had considerable difficulty determining the immediate Paleozoic outgroups of modern lissamphibians, but two major groups of early tetrapods have been identified as credible candidates, the Lepospondyli and the Temnospondyli. Lepospondyls are a morphologically diverse group of early tetrapods with little unifying morphology aside from small body size. Temnospondyls typically all share a common bauplan but exhibit a substantial disparity of body sizes, although putative lissamphibian outgroups within Temnospondyli are also small-bodied (Fröbisch and Schoch, 2009; Pérez-Ben et al., 2018). Phylogenetic support for the two hypotheses has traditionally been roughly within a statistical margin of error (Ruta and Coates, 2007; Marjanović and Laurin, 2019) with differing implications for both pattern of lissamphibian body plan assembly and timing of the origin of the tetrapod crown group.

Traditionally, both temnospondyls and lepospondyls have been considered early diverging tetrapod clades that originated as part of an early Carboniferous tetrapod diversification. Because of a poor vertebrate record in the earliest Carboniferous, the earliest representative of this diversification has traditionally been the lepospondyl *Lethiscus stocki* (Figure 3D; Anderson et al., 2003; Benton et al., 2015), which would be considered a crown tetrapod under either major lissamphibian origins hypothesis (a stem-amniote under the temnospondyl hypothesis or a stem-amphibian under the lepospondyl hypothesis, Figures 1B,C), and *Lethiscus stocki* has been therefore conveniently recommended by paleontologists as the appropriate node calibration for the tetrapod crown (Benton and Donoghue, 2006; Benton et al., 2015). However, recent description of tetrapod faunas from earliest Carboniferous fossil deposits (Anderson et al., 2015; Clack et al., 2017, 2018) has identified many taxa within this early diversification that were thought to be characteristic of later Carboniferous or Permian faunas, demanding a more careful consideration of which of these Carboniferous forms belong to the crown group. Recent reanalysis of *Lethiscus* has shown that such reconsideration is not only justified but also necessary, as it shares a number of anatomical features with definitive Devonian stem-tetrapods not seen in the Carboniferous radiation (Pardo et al., 2017b). The earliest temnospondyl, *Balanerpeton* (Milner and Sequeira, 1993) from the Viséan (∼335 MA) of East Kirkton, Scotland, by contrast, is widely accepted in its identification and establishes the temnospondyl (sensu strictu, independent of the placement of colosteids) portion of this dichotomy.

There is some uncertainty in the overall relationships of tetrapod taxa that make up this Carboniferous radiation, but there are some broad patterns. Traditionally, the least inclusive clade including temnospondyls and modern amniotes is thought to include most if not all Carboniferous tetrapod taxa (Ruta et al., 2003; Ruta and Coates, 2007), regardless of whether amphibians originated within Temnospondyli or Lepospondyli (Figures 1A,B). Specifically, this clade is thought to include the Embolomeri (Figure 3C), a group of large to very large predatory tetrapods, which are typically considered to be more closely related to amniotes than temnospondyls but less closely related to amniotes than lepospondyls (Ruta et al., 2003). It sometimes also includes the Colosteida (Figure 3B), a group of aquatic elongate-bodied forms that appears as the sister group of Temnospondyli in some analyses. Thus, in most common formulations, the Temnospondyl hypothesis extends the age of the tetrapod crown group to the age of the oldest embolomere or colosteid, whereas the Lepospondyl hypothesis set the age of the tetrapod crown at the appearance of the earliest lepospondyl, the aïstopod *Lethiscus stocki*, from the middle Visean of Scotland (Marjanović and Laurin, 2013). Because fragmentary embolomere-like and colosteid-like limb elements have been recently reported from the early Tournaisian of Nova Scotia (Anderson et al., 2015), the Temnospondyl hypothesis may implicitly support an age of the tetrapod crown group at the Devonian-Carboniferous boundary.

Furthermore, within a Temnospondyl hypothesis framework, some variation in estimated age of the crown also depends on the phylogenetic position of two problematic taxa: the fragmentary Devonian tetrapod *Tulerpeton curtum* and the Whatcheeriidae, a group of animals widespread in the lower Carboniferous (Lombard and Bolt, 1995; Clack, 1998; Warren, 2007) but present in the uppermost Devonian (Daeschler et al., 2009; Olive et al., 2016). Both *Tulerpeton* and the whatcheeriids have generally been found on the tetrapod stem in most analyses (Ruta et al., 2003; Clack et al., 2017; Pardo et al., 2017b; Marjanović and Laurin, 2019) but appear on the amniote stem in a subset of studies (e.g., one of the three trees reported by Clack et al., 2017). *Tulerpeton* consists primarily of a single articulated but headless holotype (Figure 3A). Because the majority of anatomical data used in phylogenetic analyses are cranial (Ahlberg and Clack, 1998; Pardo et al., 2017b), the phylogenetic placement of *Tulerpeton* depends largely on less-studied anatomy and more general anatomy of the limb elements (Lebedev and Coates, 1995), in particular the unusually shaped humeral and cylindrical femoral shafts. Conversely, *Tulerpeton* exhibits prominent adductor blades on the femur (shared with *Ichthyostega* and *Acanthostega*) and a polydactylous manus (shared with *Acanthostega* and *Ichthyostega*). Whatcheeriids, best typified by *Whatcheeria deltae* from the Visean of Iowa, United States, but also including *Pederpes finneyae* from the Tournaisian of Scotland and *Ossinodus puerhi* from the Visean of Australia, are somewhat better-known than *Tulerpeton*. *Whatcheeria* was first compared with embolomeres, considered by some to be stem-amniotes, on the basis of the deep skull and short postorbital skull table (Lombard and Bolt, 1995), although the authors acknowledged that most of these embolomere-like features only weakly support this placement. However, *Whatcheeria* also preserves many features that are either plesiomorphic or are found only in the stem-tetrapod *Ichthyostega*, including large triangular flanges on the ribs and a buccohypophyseal foramen (Bolt and Lombard, 2018). In resolving the phylogenetic relationships of both of these taxa, there are deep conflicts between character complexes and treatments. These conflicts have major implications for the timing of tetrapod origins: although inclusion of one or more of these taxa in a more derived position that temnospondyls suggests no change in the timing of crown tetrapod origins under the Lepospondyl hypothesis, this would suggest a very deep origin of tetrapods under the Temnospondyl hypothesis, emphasizing a central need to resolve the lissamphibian origins debate in order to inform deeper node calibrations within the tetrapod tree.

This debate itself depends on two major features of tetrapod phylogeny: a monophyletic Lepospondyli that is closely related to amniotes and an early divergence of Temnospondyli within the Late Devonian or early Carboniferous radiation. It increasingly appears that the former is not a settled feature of early tetrapod phylogeny. Recent redescription of a number of recumbirostrans, a clade of lepospondyls part of the previously recognized Order Microsauria (Carroll and Gaskill, 1978), has shown surprisingly reptile-like morphology of the braincase, suspensorium, and lower jaw (Pardo et al., 2015; Szostakiwskyj et al., 2015; Pardo and Anderson, 2016). In contrast, micro–computed tomography study of the aïstopod *Lethiscus stocki*, the earliest lepospondyl, has revealed extremely fishlike organization of the head (Pardo et al., 2017a), suggesting that the diverse morphology of lepospondyls may be a function of polyphyletic origins across the early tetrapod tree rather than a single adaptive radiation. Although this does not exclude the possibility that one lepospondyl group might represent the lissamphibian stem group, the likely polyphyly of lepospondyls means that supporters of the Lepospondyl hypothesis must specify which lepospondyl group they consider most closely related to lissamphibians and must identify node calibration dates accordingly. Regardless, it is unlikely that *Lethiscus* can remain the node calibration. With the exception of the ambiguous *Westlothiana lizzeae* (Smithson et al., 1993) and *Kirktonecta* (Clack, 2011b) and some fragmentary fossils attributed to microsaurs from the Serpukhovian of Goreville, Kentucky (Lombard and Bolt, 1999), there are few Mississippian lepospondyls aside from aïstopods and adelogyrinids, both of which are unlikely to be lissamphibian stem groups. The first unambiguous members of the remaining lepospondyl groups (microsaurs, nectrideans, lysorophians) are earliest Pennsylvanian in age and from the same localities as the earliest amniotes.

The broader patterns of early tetrapod phylogeny may be under dispute as well. In particular, several new lines of evidence suggest that the early Carboniferous tetrapod diversification may be limited to stem-group tetrapod lineages and that the divergence of lissamphibians and amniotes may be substantially more recent, even under the Temnospondyl hypothesis. These lines of evidence come from restudy of colosteids and embolomeres themselves and suggest an emergence of both taxa within the Devonian radiation of early tetrapods, prior to tetrapod terrestrialization. In colosteids, this has come from new comprehensive studies that have found that the colosteid skull and jaw retain many bones lost in more advanced taxa and that similarities with temnospondyls are likely superficial (Bolt and Lombard, 2001, 2010). In contrast, studies addressing embolomere anatomy have remained relatively restricted in anatomical scope. Embolomeres have often been considered early representatives of the lineage leading to amniotes, based on the deep narrow skull and large size of the Meckelian foramen in the lower jaw, among other features (Carroll, 1970). However, recent work has identified substantial conflicts between anatomical suites, suggesting that reconsideration of this scenario is necessary. Most notably, Clack (2011a) identified the presence of dermal fin rays (lepidotrichia) and bony supports (supraneural radials) in the caudal fin of a partial embolomere tail and likely presence of supraneural articulations in other more complete embolomeres, but did not address whether this would suggest an earlier divergence of embolomeres within tetrapods or a reversal in this one species. Pardo et al. (2018) drew several comparisons between the skull and braincase of aïstopods and embolomeres and identified evidence for articulation between the dorsal branchial skeleton and the otoccipital regions of both taxa. As a dorsal branchial skeleton is thought to be retained only through the fin-to-limb transition in tetrapods (Coates and Clack, 1991), this would provide further evidence for placing embolomeres on the tetrapod stem, regardless of one’s hypothesis of lissamphibian origins. Indeed, recent phylogenetic treatment of endocranial data from these and other early tetrapods has found increased evidence for a closer relationship between temnospondyls and amniotes to the exclusion of both colosteids and embolomeres (Pardo et al., 2017a), possibly indicating a much more exclusive crown group under the temnospondyl hypothesis.

It is important to note that the proceeding discussion relates to only one view of overall tetrapod phylogeny, the tree of Ruta et al. (2003) and Ruta and Coates (2007), but other hypotheses similarly struggle with these issues. Another hypothesis, that of Smithson (1985), posits a deep divergence between reptiles and lissamphibians, with reptiles descending from a long lineage from embolomeres to anthracosaurs called Reptilomorpha (note, this concept differs from that defined phylogenetically by Laurin, 2001; Vallin and Laurin, 2004). Given this hypothesis (which has not been supported by the largest computer assisted analyses conducted to date but has had some support from more limited treatments, such as Ruta and Clack, 2006), the split between reptilomorphs and batrachomorphs (the lissamphibian stem group) would be placed at least into the Viséan and possibly extend possibly into the Devonian, should *Tulerpeton* (not included in the analysis of Ruta and Clack, 2006, which they state was “not intended as an exhaustive investigation of early tetrapod relationships” [p. 49]) prove to be an embolomere.

## Node Maxima: Is the Early Tetrapod Record Complete Enough to Rely on Node Calibrations?

Assignment of maxima (hard or soft) depends on confidence in the quality of the fossil record. Reviews suggesting hard and soft maxima for major tetrapod clades (e.g., Benton et al., 2015) generally point to faunas entirely lacking any members of these groups. Assignment of hard maxima must contend with the understanding that absence of evidence is not evidence of absence, but that continued absence after sufficient sampling may provide a degree of confidence in absence. Sampling of a crown group fossil within a fossil collection requires that four criteria are met:

(1) the crown-group animal has the same (or better) probability of being preserved in the fossil localities sampled in comparison with outgroups;
(2) known fossil localities sample the kind of local habitats where the crown-group animals lived and died;
(3) known fossil localities sample the biogeographic provinces where crown-group animals were distributed; and
(4) sampling effort is sufficient within an interval and region to assume that the crown-group animal would have been found if it were present, which itself is a function of species prevalence (e.g., Hedman, 2010).

If all four of these conditions are met and no members of the crown group are identified, it becomes more reasonable to infer that the crown group may not have originated by a specific interval. This presents a substantial challenge: when is a representative of a crown group absent from a collection or fauna because it did not exist at the time, and when is it absent from a collection or fauna because one or more of these four conditions has not been met?

Benton et al. (2015) provide three distinct justifications for soft maxima for the three major tetrapod clades reviewed here. The soft maximum for crown amniotes is set at 332.9 Ma based on the absence of crown amniotes at the East Kirkton locality within the Visean of Scotland. The soft maximum for the amphibian crown group is set at the base of the Middle Permian (272.8 Ma) and based on the absence of definitive stem amphibians in the Middle and Upper Permian rocks of Russia, China, and South Africa. The soft maximum for crown tetrapods is set at the middle Tournaisian (351 Ma) based on the presence of the whatcheeriids *Pederpes finneyae* and *Whatcheeria deltae* in Scotland and North America, respectively.

Already it should be apparent that some of these soft maxima are substantially younger than the age of the oldest member of the crown group according to different phylogenetic hypotheses. For example, if we accept that the temnospondyl *Gerobatrachus hottoni* is in fact a stem-group batrachian following Anderson et al. (2008) and Maddin et al. (2012), then the hard minimum age of the amphibian crown group must be the age of *Gerobatrachus*, which is no younger than 272.8 Ma and likely closer to 276.2 Ma, the maximum age of the Tubb Sandstone inferred by U-Pb dating of detrital zircons (Liu and Stockli, 2020). The type locality of *Gerobatrachus hottoni* is in the informal “Cedar Top Sandstone” unit of the Middle Clear Fork Group (R. Hook, pers. comm.), which sits below the Tubb Sandstone (Nelson et al., 2013a, b). The recent suggestion by Pardo et al. (2017b) that the amphibian crown group may be even more inclusive would set the hard minimum at a substantially older age (∼315 Ma) over 40 million years older than the soft maximum of Benton et al. (2015). Disagreements exist in both directions for the age of the tetrapod crown group; some studies of early tetrapod phylogeny suggest that the hard minima for the tetrapod crown group is older than the soft maximum offered by Benton et al. (2015), although recent phylogenetic analyses relying on more sophisticated treatment of endocranial anatomy suggest a much younger age of the tetrapod crown more generally (Pardo et al., 2017a, 2019; Pardo and Mann, 2018).

Additionally, the soft maxima suggested for major tetrapod clades may conflict with the criteria outlined above. These conflicts are themselves a combination of overlapping deficiencies in the vertebrate fossil record. These deficiencies are a product of systematic preservation and sampling biases in space and time and correspond to areas of great uncertainty in the fossil record of major tetrapod clades.

### Preservational Heterogeneity and Small Body Size

The assembly of both the amphibian crown group and the amniote crown group are thought to have largely occurred at small body sizes (Carroll, 1982; Laurin, 2004; Kemp, 2007; Pérez-Ben et al., 2018), although the overall pattern of body size evolution in these clades is under some debate (Didier et al., 2019). Skeletal material from small vertebrates degrades more quickly than bones of larger vertebrates and is preferentially lost from the record (Behrensmeyer et al., 1979). This creates a set of related patterns that have the potential to preferentially deplete the fossil record of early members of major tetrapod crown groups. This is apparent in the particularly large gaps in the caecilian fossil record (Evans and Sigogneau-Russell, 2001; Jenkins et al., 2007). First, this would suggest that early members of the amphibian and amniote crown groups may be expected to be absent across much of the early tetrapod fossil record even if they were present at the time. Furthermore, this would suggest that what remnants of early crown-group amphibians, amniotes, and perhaps even crown tetrapods may also be preferentially more degraded, reducing the ability of specialists to identify isolated elements of small-bodied early tetrapods to higher taxon. Finally, this would suggest that early representatives of both amphibian and amniote stem groups (and potentially crown groups) can be expected to reflect distribution of localities with exceptional preservation.

Exceptional preservation in the fossil record is itself a function of several properties of specific local or regional depositional environments. Rapid burial in an anoxic reducing environment is generally a prerequisite for exceptional preservation and is most typical of environments with standing water. This is the case for the majority of exceptional vertebrate-bearing fossil localities across the late Paleozoic, which include anoxic organic-rich oxbow lakes (e.g., Hook and Baird, 1986; Clarkson et al., 1993), largely anoxic graben lake deposits, and shallow brackish lagoons (e.g., Clements et al., 2019). Aside from sites of exceptional preservation, small vertebrate skeletons may be concentrated but remain relatively undisturbed in very specific circumstances, such as within fissure fill deposits such as the Fort Sill locality (MacDougall et al., 2017) and the classic Joggins lycopod stump localities (Falcon-Lang et al., 2006). These latter types of localities are exceedingly rare and preserve a unique fauna, but the deposition in anoxic lacustrine or estuarine systems tends to be repeated where similar environmental conditions are present (Baird et al., 1985). This means that trends in both regional paleoenvironment and global paleoclimate may bias discovery probability in a given region or interval.

What does this mean for the probability of discovery of small-bodied tetrapods across the Late Paleozoic? Although this has not been investigated for the entire Paleozoic tetrapod record, regional trends have been investigated for the interval spanning from 315 to 272 Ma and found that the vertebrate record samples these sorts of environments well only in the late Carboniferous, and this sampling of these environments reflects regional variation in climate change across the late Paleozoic (Pardo et al., 2019). Such environments are poorly sampled outside of Europe and North America in this interval, and in fact are essentially completely unsampled in the “classic” Upper Permian sequence of South Africa until the earliest Triassic. This presents a dual challenge in discovering the earliest representatives of the amniote and amphibian crown groups. First, the earliest amniotes were certainly highly terrestrial (Carroll, 1964; Laurin and de Buffrénil, 2016), and it appears that the earliest amphibians may have been as well (Pardo et al., 2019), and therefore lived in habitats that may have been spatially separated from ideal preservational environments. This means that early amniotes and amphibians are likely rare even among rare vertebrate fossils and will likely not be seen in localities without extensive worker effort. Second, the probability of discovery is likely limited by the abundance of these sorts of localities. Within the Lower Carboniferous, only three localities contain this level or preservation: the nearshore marine Wardie Shale (Trojan et al., 2015) and Cheese Bay Shrimp Bed (Hesselbo and Trewin, 1984), and the thermally altered lake deposits at East Kirkton (Clarkson et al., 1993). Of these, the only locality that has yielded more than a single tetrapod fossil is East Kirkton. East Kirkton also represents the only definitive Lower Carboniferous occurrences of crown tetrapods within recent phylogenetic analyses (Milner and Sequeira, 1993).

### Spatiotemporal Heterogeneity

**FIGURE 4:**
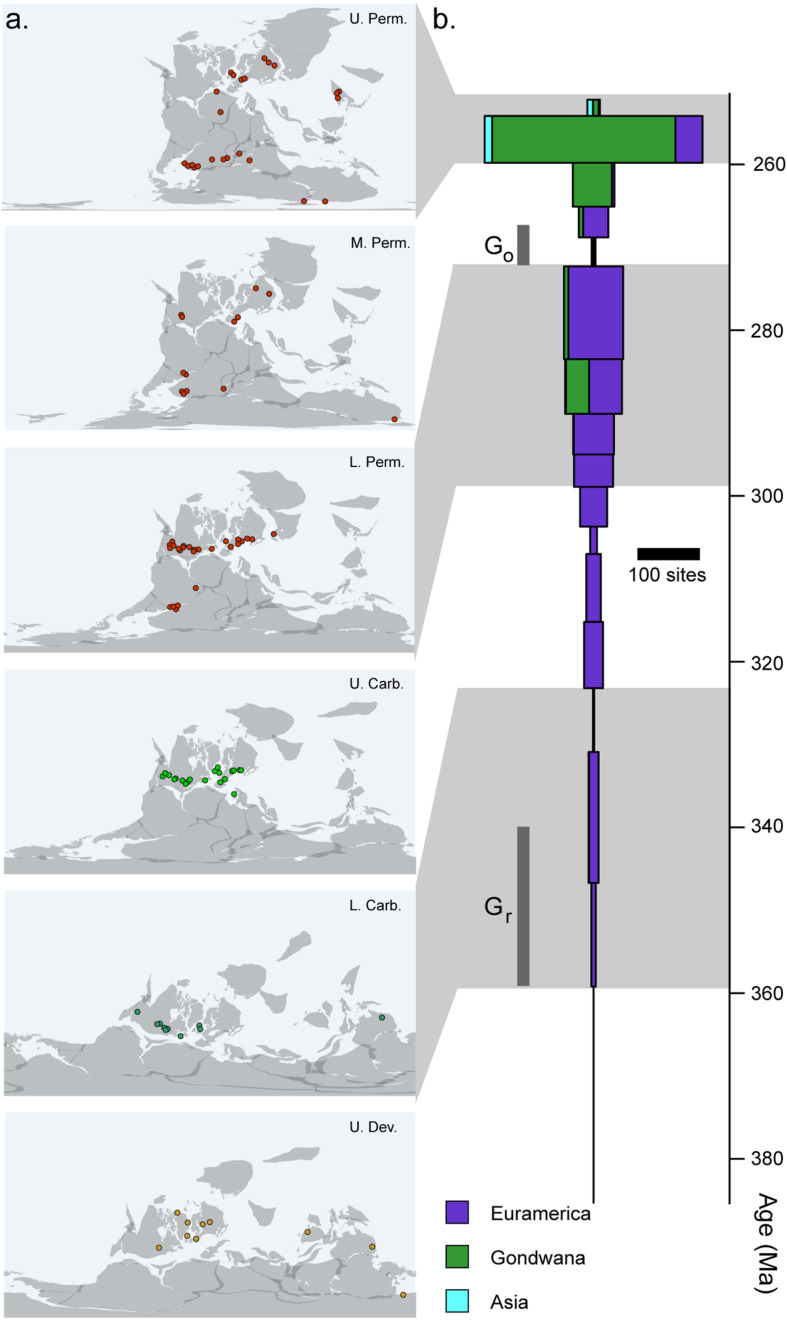
Completeness of the early tetrapod record. **(A)** Geographic distribution of tetrapod fossil localities across the late Palaeozoic. **(B)** Number of tetrapod-bearing localities recorded in the Paleobiology Database (http://paleobioDB.org) by stage, showing heterogeneity in regional and temporal sampling.

In addition to the sampling biases imposed by small body size, there are several broader patterns of the early tetrapod fossil record that may also preferentially obscure the early records of crown tetrapods, crown amphibians, and crown amniotes. In particular, the early tetrapod record is itself relatively heterogeneous in both space and time (Figure 4). The fossil record of tetrapods from the Late Devonian until the Middle Permian is almost entirely restricted to localities from North America and Europe (Milner, 1993). At the time, this represented a single continental landmass restricted to within 10 degrees of the equator (Figure 4A). A few localities exist outside of this narrow equatorial band in the Devonian, Carboniferous, and Permian, but taxonomic diversity and worker effort remain far lower in these regions than in Euramerica, particularly in Gondwana, which had not fully joined the Pangaean supercontinent until the late Carboniferous to early Permian (Ziegler et al., 1979; Stampfli et al., 2013). A robust record outside of Euramerica does not appear until the latter part of the Middle Permian (Figures 4A,B), at which point concurrent well-sampled records appear in the Karoo Basin of South Africa (Rubidge et al., 2013), the Paraná Basin of southern Brazil (Dias-da-Silva, 2012), and a series of basins across Russia and China (Olroyd and Sidor, 2017). These faunas preserve very different vertebrate communities dominated by diverse and abundant derived mammal-line synapsids not observed in Euramerica.

This would not be a substantial problem if early tetrapods did not show any substantial biogeographic patterns. However, where faunas outside of the Euramerican transect are known, they contrast in some important ways with contemporary Euramerican faunas, preserving both unique lineages and extremely early records of Late Permian taxa (Milner and Panchen, 1973; Cisneros et al., 2015). Carboniferous-Permian faunas from central Asia are dominated by seymouriamorphs (interpreted as stem amniotes under most phylogenetic hypotheses) but completely lack representatives of classic Euramerican taxa until well into the Middle Permian (Reisz and Laurin, 2001). Interestingly, central Asia appears to be the epicenter of another putative stem-amniote lineage, the Chroniosuchia, which appears to be completely absent from the Carboniferous-Permian transition of Euramerica, as well as later sequences across Gondwana (Golubev, 1998; Witzmann et al., 2008). Conversely, new localities from the Carboniferous-Permian transition of Gondwana seem to preserve a tetrapod community that is roughly similar to Euramerican faunas but include unique Permo-Triassic-like components, including rhinesuchid stereospondyls and advanced lungfishes (Cisneros et al., 2015), further hinting at important biogeographic patterns, either regional endemism or differences in paleoenvironments. These biogeographic patterns are not restricted to the fossil record; distribution of extant amphibians suggests a clear Gondwanan origin of crown-group caecilians and Laurasian origin of salamanders, with modern representatives essentially restricted to these regions (recently summarized in Pyron, 2014). Given this substantial provincialization, it would seem at least possible that the origin of some major tetrapod clades may have occurred in a biogeographical province not currently represented in the tetrapod record. The most obvious candidate group would be amniotes, which are already highly diverse at the time of their first appearance and are not preceded by an unambiguous stem group. An origin of amniotes in a Central Asian epicenter would appear plausible and has been suggested at least obliquely in qualitative studies of the tetrapod record at this time (Milner and Panchen, 1973).

Furthermore, the early tetrapod record is punctuated by several key intervals of minimal sampling of the tetrapod record (Figure 4B). Two of these are particularly noteworthy: an 18-million-year interval in the lower Carboniferous, spanning from the Devonian-Carboniferous boundary (358.9 Ma) until the middle Viséan (∼330.9 Ma), and a second within the first half of the middle Permian (272–265 Ma). The former is generally referred to as Romer’s Gap and likely coincides with the origin of the tetrapod crown group, whereas the second, referred to as Olson’s Gap, coincides with a major faunal turnover between Carboniferous-Permian transition faunas dominated by archaic tetrapods and early amniotes and Upper Permian faunas dominated by diverse therapsid-grade stem mammals, but also spans an interval that may represent the assembly of distinct lissamphibian body plans (Marjanović and Laurin, 2007; Anderson et al., 2008; Pardo et al., 2017a). It has been suggested previously that this transition, at least among synapsids, represents a physiological shift in response to the rapidly changing environment (Kemp, 2006). Although some work has been done in recent years to bridge these sampling gaps (Smithson et al., 2012; Anderson et al., 2015, Clack et al., 2017), these remain relatively unexplored intervals, and it is not possible to assess at this time whether the absence of identified fossils of key informative taxa (Mississippian crown-group tetrapods, middle Permian crown-group lissamphibians) represents a real absence from these faunas. Further intervals are also substantially undersampled in addition to these historical “gaps.” This extends throughout the Lower Carboniferous (Figure 4B), where sampling effort is not only very poor but is extremely geographically restricted (Figure 4A). Given that this interval appears to contain, at the very least, the origin of both crown-group tetrapods and crown-group amniotes, confidently applying limits to age estimates for these nodes is likely impossible.

## Discussion and Recommendations

Attempts to establish *a priori* constraints for major tetrapod clade ages must contend with two parallel problems: there is little agreement on the inclusiveness of these clades, and the early tetrapod record is so unevenly sampled that we cannot assume representative sampling of early members of these clades. The result is that two of the three nodes assessed here exhibit substantial variation in *a priori* calibration ages based on phylogenetic hypothesis, with a range of credible estimates spanning over 30 million years for *a priori* calibrations of the tetrapod crown and over 70 million years for *a priori* calibrations of the lissamphibian crown. Recent work has suggested that the earliest representatives of the tetrapod crown group may be substantially younger than previously thought, whereas new fossils and hypotheses may support substantially older calibration ages for the lissamphibian crown group than previously appreciated. These depend on three major points of phylogenetic disagreement (amphibian origins within early tetrapods, caecilian origins within total-group amphibians, and delimitation of the tetrapod crown group) that will likely remain under debate for some time in the future, but workers calibrating deep nodes in the tetrapod tree should be prepared to take these ages into account.

Conversely, the *a priori* constraints on the age of the first representatives of the amniote crown are relatively robust to phylogenetic disagreement. However, the earliest amniotes appear after a long interval of poor sampling, and early members of the amniote total group show extremely poor stratigraphic concordance, with members of the amniote stem appearing millions of years later than the earliest crown-group amniotes. One explanation for this problem is that the faunas in which amniotes originated are unsampled within the early Carboniferous. This can be attributed to multiple factors: (1) that early Carboniferous localities heavily sample aquatic habitats, but only poorly sample dryland terrestrial environments (e.g., Pardo et al., 2019); (2) that amniotes originated in a biogeographic region outside of and with limited connectivity to Euramerica prior to the Late Carboniferous; (3) or a combination of both explanations. Such a hypothesis would not necessarily be limited to amniotes; crown tetrapods in general seem to have appeared abruptly at the end of the Early Carboniferous within a relatively brief 20-million-year interval, with high levels of terrestriality seen across the tetrapod crown group in general (Pardo et al., 2019). One suggested location for this biogeographic province would be the Kazakh plate that now forms much of central Asia and that is home to a uniquely diverse putative stem amniote assemblage in the early Permian (Milner and Panchen, 1973), but this evidence remains highly circumstantial, given that no early amniotes are known from this province and the seymouriamorph-dominated assemblages appear to be younger than the earliest amniote-dominated assemblages, such as Joggins, from Euramerica. There is little direct evidence for any such phylogeographic structure of Carboniferous tetrapod assemblages without new sampling from the Carboniferous of Gondwana and Asia, as well as more aggressive sampling within the interval roughly between 320 and 340 Ma.

This problem might be resolvable if molecular clock estimates converge on a tight estimate of the origins of major tetrapod clades with tight correspondence to a subset of hypotheses. This has been argued from both the molecular (San Mauro, 2010) and paleontological (Marjanović and Laurin, 2007) perspectives. However, we find no such tight correspondence. In fact, the dispersion of molecular clock estimates broadly compares with the dispersion of possible calibration dates, in that the estimated ages of the tetrapod and amphibian crown groups are difficult to constrain, whereas the amniote crown group is more tightly constrained. As most molecular clock analyses have used the node calibrations of Benton and Donoghue (2006) and Benton et al. (2015) for the tetrapod and amphibian crown groups, it seems likely that the uncertainty is a function of the poor early tetrapod (and amphibian) fossil record, which interacts with variation in taxonomic and molecular sampling and model parameterization to produce highly volatile estimates.

Recommendations for best practices in calibrating nodes in molecular clock studies have been previously made by Parham et al. (2012), with a focus on ensuring that calibration ages are replicable by tying the age to a specimen and stratigraphic horizon. However, these recommendations generally do not provide guidance for dealing with the problems we have identified here in calibrating nodes in the Palaeozoic and earlier. With this in mind, we urge that those completing studies calibrating deep tetrapod nodes, as well as other deep nodes, to keep the following in mind:

(1) Compendia of node age calibrations, such as those of Benton and Donoghue (2006) and Benton et al. (2015) may misrepresent confidence in node age calibrations from the Palaeozoic by understating disagreement between specialists on the underlying phylogeny and even anatomy. This is particularly problematic for Palaeozoic and pre-Palaeozoic calibrations where anatomical evidence from modern representatives of clades may be scarce and where the record may be generally poor.
(2) Molecular clock studies relying on deep tetrapod node calibrations should be cognizant of disagreements in phylogenetic analyses and should try as much as possible to incorporate this uncertainty where possible. Because possible ages of the tetrapod and lissamphibian crown groups vary so much, depending on specific phylogenetic hypotheses, we strongly recommend conducting multiple independent calibrations rather than adjusting hard minima and soft maxima to include the full range.
(3) In cases where a single tree and a single set of node calibrations are used, authors must explicitly state and defend the phylogenetic hypothesis used to generate those calibrations in terms of confidence in the underlying tree and its associated hypotheses of body plan evolution. Some datasets may be easier to adopt into total evidence approaches, but differences in total number of characters, total number of fossil operational taxonomic units, or degree of taxonomic overlap with molecular datasets do not necessarily reflect confidence in the underlying topology among specialists.
(4) Application of more precise calibration approaches (e.g., tip dating and fossilized birth-death models) cannot be considered a replacement for satisfactorily resolving phylogenetic uncertainty in the origin of the tetrapod and amphibian crown groups.
(5) Tip-dating approaches should not be used as an independent assessment of the quality of priors, including node calibration priors or tree priors. Regardless of the arguments for or against the use of tip-dating methods for assessing quality of priors, the early tetrapod record is highly heterogeneous both in terms of the observed pattern of preservation and the taxonomic expectation of preservation. It is therefore likely that the tetrapod record will violate certain assumptions of tip-dating approaches unless appropriately parameterized.
(6) Early tetrapod workers need to bring their attention to undersampled intervals and regions. Establishing a tetrapod fossil record from the Carboniferous and Early Permian of Asia and Gondwana is of particular importance. Terrestrial rocks are known from these regions, in some cases preserving fossils of other vertebrate groups (actinopterygians, chondrichthyans, and dipnoans) and plants, but tetrapods from these rocks are essentially unknown.

These best practices can be applied more generally to efforts to calibrate nodes prior to the end of the Palaeozoic, as many of the same principles apply to phylogenetic problems among Palaeozoic organisms more generally (difficulty relating extant phylogenetic patterns in anatomy to earliest fossil relatives, preservational biases, temporospatial megabiases, etc.). We do caution that the particular problems we identify here with relating the early tetrapod record to the origin of major tetrapod clades may not directly correspond to problems in other groups, although conceptual similarities almost certainly exist. Workers attempting to calibrate these nodes should exercise caution and seek direct consultation with experts on relevant parts of the fossil record.

## Author Contributions

JP led the project and created the images. KL generated new data on nectrideans. JA underwrote the project and provided CT access. All authors wrote the manuscript.

## Conflict of Interest

The authors declare that the research was conducted in the absence of any commercial or financial relationships that could be construed as a potential conflict of interest.
